# Fast individual ancestry inference from DNA sequence data leveraging allele frequencies for multiple populations

**DOI:** 10.1186/s12859-014-0418-7

**Published:** 2015-01-16

**Authors:** Vikas Bansal, Ondrej Libiger

**Affiliations:** 1 Department of Pediatrics, University of California San Diego, 9500 Gilman Drive, La Jolla, 92093 CA USA; 2 Scripps Translational Science Institute, 3344 N Torrey Pines Court, La Jolla, 92037 CA USA; 3 Current address: MD Revolution, San Diego, CA USA

**Keywords:** Admixture estimation, High-throughput sequencing, Allele frequencies, Maximum likelihood, Ancestry, BFGS algorithm

## Abstract

**Background:**

Estimation of individual ancestry from genetic data is useful for the analysis of disease association studies, understanding human population history and interpreting personal genomic variation. New, computationally efficient methods are needed for ancestry inference that can effectively utilize existing information about allele frequencies associated with different human populations and can work directly with DNA sequence reads.

**Results:**

We describe a fast method for estimating the relative contribution of known reference populations to an individual’s genetic ancestry. Our method utilizes allele frequencies from the reference populations and individual genotype or sequence data to obtain a maximum likelihood estimate of the global admixture proportions using the BFGS optimization algorithm. It accounts for the uncertainty in genotypes present in sequence data by using genotype likelihoods and does not require individual genotype data from external reference panels. Simulation studies and application of the method to real datasets demonstrate that our method is significantly times faster than previous methods and has comparable accuracy. Using data from the 1000 Genomes project, we show that estimates of the genome-wide average ancestry for admixed individuals are consistent between exome sequence data and whole-genome low-coverage sequence data. Finally, we demonstrate that our method can be used to estimate admixture proportions using pooled sequence data making it a valuable tool for controlling for population stratification in sequencing based association studies that utilize DNA pooling.

**Conclusions:**

Our method is an efficient and versatile tool for estimating ancestry from DNA sequence data and is available from https://sites.google.com/site/vibansal/software/iAdmix.

**Electronic supplementary material:**

The online version of this article (doi:10.1186/s12859-014-0418-7) contains supplementary material, which is available to authorized users.

## Background

Allele frequencies at most loci in the human genome differ between populations as a result of human demographic history and genetic drift [1]. Individuals can be grouped into genetic clusters that correspond to major geographic regions using information about genotypes at multiple loci [2]. Individuals whose ancestors originated in different populations, and who are, therefore, admixed, exhibit ancestry associated with multiple different genetic clusters or populations. For example, the majority of African Americans possess 10-20% of their genetic ancestry consistent with European genetic background with the remainder of their ancestry being African [3].

Estimating the unknown admixture proportions of an individual is valuable for understanding human population history as well as controlling the rate of false associations in disease association studies by avoiding or correcting for population stratification, i.e. differences in ancestry between cases or controls [4,5]. A widely used approach to correct for population stratification is to include estimates of admixture proportions for each individual as covariates in statistical models testing for association [6].

Two types of methods have been developed for the analysis of ancestry and population structure using genetic data: model-based clustering methods such as STRUCTURE [7], FRAPPE [8] and ADMIXTURE [9], and principal component analysis (PCA) [10]. Model-based clustering methods model a population using allele frequencies at multiple loci and each individual’s genome as an admixture of alleles from different populations.

Given a fixed number of clusters (populations), *K*, these methods use an unsupervised clustering approach to simultaneously infer the allele frequencies associated with the *K* clusters and estimate the relative contribution of the *K* clusters to each individual’s ancestry. The low cost of whole-genome genotyping assays has enabled comprehensive surveys of genetic variation and these methods have been highly successful in understanding the population structure in many different human populations [11-15].

Most existing methods for analysis of admixture and ancestry have been designed to analyze population structure in an unsupervised manner. Supervised analyses of admixture can be valuable for estimating accurate admixture fractions for individuals whose ancestral history is known. For example, accurate admixture fractions for African American individuals associated with European and African ancestral populations can be obtained using ADMIXTURE and similar software only if European and African individuals are included as reference. Alexander and Lange [16] have extended ADMIXTURE to carry out supervised analysis by including genotype data for individuals who belong to predefined population clusters. However, supervised analyses of an individual’s genetic ancestry can be performed using population allele frequencies alone and does not necessarily require individual level genotype data.

Another limitation of existing methods is that these methods were designed to process data generated from genotyping arrays and require precise knowledge of the genotypes for each individual. As a result, these methods are not well suited to inferring ancestry from DNA sequence data where the genotypes may not be known precisely. As the cost of DNA sequencing has decreased rapidly, high-throughput sequencing instruments such as the Illumina HiSeq are being used to sequence large number of human genomes and disease association studies are being pursued using high-throughput sequencing instead of genotyping arrays [17]. Sequencing the entire human genome can still be too costly, and many studies perform low-depth sequencing to obtain information about variants and genotypes. For example, the 1000 Genomes project has performed low-coverage (2-4 x) whole-genome sequencing for thousands of individuals from diverse populations [18]. Other studies utilize targeted sequencing where only specific regions of the genome, e.g. the coding regions of genes, are targeted for sequencing. Interestingly, a significant fraction of the reads derived from targeted sequencing fall outside of the targeted regions. Various studies have shown that 30-50% of the reads map outside target regions [19]. Each off-target read that covers a single nucleotide polymorphism (SNP), for which reference population allele frequency information exists, is weakly informative about the genotypes of the individual, and can be used to infer ancestry.

With the increasing use of high-throughput sequencing for studies of human disease and population history, there is a need for computationally efficient methods for ancestry inference that can effectively utilize existing information about allele frequencies associated with different human populations and can work not only with genotypes but also with DNA sequence reads. Recognizing this challenge, several methods for ancestry inference from sequence data have recently been developed [20-22]. The NGSadmix method [20] essentially extends the ADMIXTURE method to work directly with sequence data using genotype likelihoods. Wang et al. [22] have developed a new method for estimation of individual genetic ancestry using analysis of sequence reads that compares each sequenced individual to a reference panel of individuals using principal-component analysis (PCA). This method simulates sequence reads for each reference individual and uses the simulated data to build a PCA map which is projected back to the original PCA space. In this paper, we propose a computationally fast method for estimating an individual’s global (genome-wide) ancestry using genotype or sequence data and pre-determined population allele frequencies associated with multiple reference populations. Our method directly incorporates the uncertainty in genotypes by working with genotype likelihoods calculated from aligned sequence reads. Our method has some similarities with NGSadmix in the use of genotype likelihoods to capture uncertainty in genotypes and with LASER in the use of a reference panel of individuals to estimate individual ancestry from sequence data. However, unlike these methods, it does not require individual genotype data for the reference populations. Using allele frequencies has two advantages: (1) it eliminates the need for the reference panel of individuals and the individual(s) being analyzed to have the same type of genetic information (genotypes vs sequence reads) and (2) the reference panel of individuals does not need to be analyzed again which leads to significant gains in computational efficiency.

Using simulated datasets, we demonstrate that our method can accurately infer admixture proportions for an individual with admixture from multiple continental populations. Using genotype data from the Human Genome Diversity Project, we show that the estimates of global genetic ancestry obtained using our method are consistent with those estimated using an existing method. Using sequence data for admixed individuals from the 1000 Genomes Project, we demonstrate that the admixture estimates are high concordant between whole genome sequence data and exome data. In addition, our technique compares very favorably with existing methods in terms of computation time. This allows us to extend our method to estimate a parsimonious set of admixture coefficients using an iterative approach.

## Methods

Previous methods for model-based ancestry analysis [7-9] perform an unsupervised analysis of the ancestry of multiple individuals and jointly estimate allele frequencies for *K* (where *K* is user-defined) ancestral populations and the relative contribution of each ancestral population to each individual’s genome. In contrast, our focus is on estimating the ancestry for a single individual using information about allele frequencies at a large number of loci for multiple reference populations. The allele frequencies for the reference populations can potentially be obtained from previous unsupervised admixture analysis of individuals from different human populations. Given an individual’s genotypes at these loci, our goal is to estimate the admixture coefficients for each population, i.e. the fraction of the individual’s genome that is derived from that population. We propose to estimate the admixture coefficients using the maximum likelihood method.

**Likelihood model for admixture coefficients:** We assume that all polymorphic sites are bi-allelic. Given a SNP with two alleles *a* and *b*, a diploid individual can have one of three possible genotypes: *aa*, *ab* and *bb*. We represent the genotype *G*_*i*_ for an individual at SNP *i* as the number of *a* alleles (0,1 or 2). Let *q*_*ij*_ denote the allele frequency of the *a* allele at the *i*-th SNP in population *j*. Given *k* reference or ancestral populations with known allele frequencies, let *a*_*j*_ represent the admixture proportion for the *j*-th population and *A*=[*a*_1_,*a*_2_,…,*a*_*k*_] be the vector of admixture coefficients. We define $f_{i} = \sum _{j=1}^{k} q_{\textit {ij}}a_{j}$ as the weighted allele frequency at SNP *i* given the allele frequencies and admixture proportions. Then, assuming Hardy-Weinberg equilibrium (HWE), the probability if observing the genotype *G*_*i*_ at site *i* is:

(1) $$ p(G_{i} |\, f_{i}) = \left\{ \begin{array}{lll} {(1-f_{i})}^{2} && \text{if}~~ G_{i} = 0 \\ 2 f_{i} (1-f_{i}) && \text{if}~~ G_{i} = 1 \\ {f_{i}}^{2} && \text{if}~~ G_{i} = 2 \end{array}\right.  $$

For a given vector of admixture proportions, the log-likelihood of the observed genotypes *g* for an individual can be defined as:

(2) $$ L(A) = \sum_{i=1}^{n} \text{ln} (Pr(G_{i} = g_{i} | \,f_{i}))  $$

where *g*_*i*_ is the observed genotype at site *i*. The above likelihood can be also be written as a function of the genotype at each site as

$$L(A) = \left[ \sum_{i=1}^{n} g_{i} \text{ln}(f_{i}) + (2-g_{i})\text{ln}(1-f_{i}) \right] + C $$

where *C* is a constant.

The above formula assumes that all SNPs are independent or in linkage equilibrium with each other. In practice, SNPs can be pruned to reduce the linkage disequilibrium (LD) between the markers [9]. Given the matrix of allele frequencies *q*_*ij*_ (1≤*i*≤*n* and 1≤*j*≤*k*) for *k* populations, our goal is to determine the vector *A*=[ *a*_1_,*a*_2_,…,*a*_*k*_] of admixture proportions that maximizes *L*(*A*) subject to the constraints *a*_*j*_≥0 and $\sum _{j} a_{j} = 1$.

### Maximizing the likelihood using the BFGS method

The likelihood function defined above is identical to the likelihood function used in previous methods [8,9] to update the admixture proportions given the allele frequencies. Our goal is to develop a computationally fast method for optimizing the likelihood function. The constraints on the admixture proportions (*a*_*j*_≥0 and $\sum _{j} a_{j} = 1$) make it difficult to utilize standard optimization techniques. ADMIXTURE uses sequential quadratic programming combined with a quasi-Newton acceleration method to optimize the likelihood function. We utilize the Broyden-Fletcher-Goldfarb-Shanno (BFGS) method to optimize the likelihood function. The BFGS algorithm [23] is a popular quasi-Newton method for solving non-linear optimization problems that utilizes the first derivatives of the likelihood function and approximates the Hessian matrix of the second derivatives.

The constraint $\sum _{j} a_{j} = 1$ can be addressed by replacing *a*_*j*_ with $\frac {a_{j}}{S(a)}$ in the log-likelihood function where *S*(*a*) denotes the sum of the admixture coefficients. This corresponds to scaling the individual admixture coefficients by their sum. The first derivates of the likelihood function can be calculated as:

$$\frac{\partial L(A)} { \partial a_{j}} = \sum_{i=1}^{n} \left[ \frac{g_{i} q_{ij}}{f_{i}} + \frac{(2-g_{i})(1-q_{ij})}{S(a) -f_{i}}\right] - \frac{2n}{S(a)} $$

To optimize the log-likelihood function, we utilized the open source implementation of the L-BFGS-B algorithm [24]. This method can handle simple box constraints required for our optimization problem (0≤*a*_*j*_≤1 for each admixture coefficient).

### Genotype likelihoods for sequence data

In the previous section, we assumed that high quality genotypes determined via genotyping arrays are available. However, it may not be possible to determine an individual’s genotypes with high precision from sequence data, especially if the depth of coverage is low. For each SNP, the information about the unobserved genotypes that is contained in the aligned reads covering the SNP can be summarized using genotype likelihoods. These genotype likelihoods correspond to the probability of observing the sequence reads conditional on the genotype at the site. Once the sequence reads have been aligned to the genome, we can determine the genotype likelihoods for each potential genotype at each site of interest using the base quality values of the individual reads. Several methods for calculation of genotype likelihoods have been proposed in the context of SNP calling from high-throughput sequence data [25-27]. We adopt an approach that is similar to these models. Let $\mathcal {R} = \{ R_{1},R_{2},\ldots R_{n}\}$ represent the set of aligned reads covering a SNP. Let *a* and *b* be the two alleles at this position.

Assuming independence between sequencing errors from multiple reads, we can define the genotype likelihoods as:

(3) $$ {\begin{aligned} Pr\left({\mathcal{R}}| G_{i} = g\right) =&\; \prod_{j,R_{j} = a} \left\{ r(1-e_{j}) + (1-r)e_{j} \right\}\\ & \times \prod_{j,R_{j} = b} \left\{ (1-r)(1-e_{j}) + {re}_{j}) \right\} \end{aligned}}  $$

where *g*=(0,1,2) is the number of *a* alleles and $r = \frac {g}{2}$ is the probability of sampling the chromosome with the ‘a’ allele. This assumes equal probability of sampling the *a* and *b* for individuals who are heterozygous. For sequence data, the probability of sampling the reference allele can be slightly greater than 50% due to mapping bias. However, this should not significantly affect the estimation of the admixture coefficients. The sequencing error probability, *e*_*j*_, can be estimated using the corresponding base quality value *q*_*j*_ as $10^{-0.1 \times q_{j}}\phantom {\dot {i}\!}$. With these definitions, we can define the log-likelihood *L*(*A*), i.e. the log of the probability of observing the sequence reads conditional on the admixture proportions *A* as:

(4) $$ L(A) = \sum_{i=1}^{n} \text{ln} \left[ \sum_{g=0}^{2} Pr({\mathcal{R}}_{i} | G_{i} = g) Pr(G_{i} = g | A) \right]   $$

where ${\mathcal {R}}_{i}$ is the set of aligned reads covering the site *i*.

### Parsimonious estimation of admixture coefficients

Given multiple reference populations, the maximum likelihood approach finds the admixture coefficients for each population that maximize the given likelihood function. Populations with a non-zero admixture coefficient are likely to contribute to the individual’s genotypes. However, in the presence of a large number of reference populations, some of which are closely related, it can be difficult to reliably estimate which populations contribute significantly to an individual’s ancestry. Imprecise allele frequency estimates due to incomplete sampling or the absence of correct parental populations can also result in non-zero admixture coefficients associated with populations that do not actually contribute to the individual’s genetic ancestry. One approach to identifying the populations that contribute significantly to the individual’s genetic ancestry is to estimate standard errors for each estimated admixture coefficient using a bootstrap approach. The ADMIXTURE method [9] uses a block bootstrap to estimate standard errors. However, this is computationally demanding since the likelihood maximization needs to be performed for several hundred resamples. We implemented a simple but rigorous approach to determine a parsimonious set of admixture coefficients for an individual by iteratively removing population(s) for which a non-zero admixture coefficient does not improve the model fit significantly. This method is analogous to the backward elimination method for variable selection. We find the population for which setting the admixture coefficient to zero does not reduce the best-fit likelihood significantly using the likelihood ratio statistic. The admixture coeffcient for this population is fixed to be 0 and this procedure is repeated iteratively. A description of the method is as follows:

1. Calculate the maximum likelihood estimate for the admixture coefficients *A*
2. For each population *j* with a non-zero admixture coefficient, calculate *δ*_*j*_=*L*_*max*_−*L*_−*j*_ obtained by calculating the maximum likelihood fit with the *j*-th admixture coefficient constrained to be 0
3. determine the population *p* with the smallest value of *δ*_*j*_
4. Set for admixture coefficient *p* to be 0 if *δ*_*p*_<*T* where *T* is a threshold based on the likelihood ratio test
5. Repeat Steps (2)-(4) until possible

The threshold *T* can be chosen according to the desired level of parsimony in the admixture coefficients. We use a threshold value of *T*=5.414 which corresponds to a p-value threshold of 0.001 using the chi-square distribution with one degree of freedom.

### Estimating ancestry from pooled sequence data

High-throughput sequencing of targeted genomic loci in large numbers of cases and controls is an effective approach for identifying rare genetic variants that affect risk for disease. Although next-generation sequencing technologies have the throughput to generate enough reads for thousands of individuals, the cost of preparing individual DNA sequencing libraries prior to sequencing limits the number of individuals that can be sequenced. A cost-effective approach for sequencing thousands of individuals is to pool DNA, in equi-molar proportions, from multiple individuals together to form pools and sequence the pools, instead of individuals [28]. This pooled sequencing approach has been used successfully to identify disease associated rare variants for a number of complex diseases: type 1 diabetes [29], inflammatory bowel disease [30], rheumatoid arthritis [31] and anorexia nervosa [32].

DNA pooling based association studies, similar to standard association studies, also require some way of correcting for population stratification. If genotype data from whole-genome arrays or at ancestry informative markers is available for each individual, this can be used to identify outlier individuals and exclude them from the pooled sequencing. However, generating individual level genotype data is costly and reduces the cost effectiveness of pooling based association studies. Therefore, a method that can estimate the average ancestry of each pool directly from the sequence reads would be valuable. The pooled admixture coefficients can be used to remove pools with very different ancestry compared to other pools from the association analysis. In addition, the admixture coefficients can be used as covariates in association analysis thereby accounting for population stratification. With this motivation, we extended our method to work with pooled sequence data derived from high-throughput sequencing of ‘artificial’ DNA pools derived by pooling DNA in equal proportions from multiple individuals.

Similar to diploid individuals, we represent the genotype *G*_*i*_ of a pool as the number of ‘a’ alleles or chromosomes at this site. Thus, if the pool has *p* diploid individuals, the number of potential pooled genotypes at a bi-allelic site is 2*p*+1. Due to errors in DNA quantification, there is likely to be some variance in the proportion of each individual’s DNA in a pool. Kim et al. [33] used a gamma distribution to model the variance in the DNA proportions from each individual in a pool. However, it is difficult to estimate the proportions without individual genotype data [34]. For ancestry assessment, it is a reasonable approximation to assume that each individual contributes equal amount of DNA to a pool.

Given the aligned sequence reads for each pool, we can calculate the genotype likelihoods $Pr({\mathcal {R}} | G_{i} = g)$ (0≤*g*≤2*p*) as follows:

$$Pr({\mathcal{R}}| G_{i} = g) = \prod_{j,R_{j} = a} f_{j} \prod_{j,R_{j} = b} (1-f_{j}) $$

where

$$ f_{j} = \frac{g}{2p}(1-e_{j}) + \left(1-\frac{g}{2p} \right)e_{j} $$

These pooled genotype likelihoods can then be used to calculate the log likelihood *L*(*A*) as defined in equation 4.

## Results and discussion

### Reference populations and allele frequencies

The HapMap 3 data set [12] includes 1,397 individuals from 11 different populations that have been genotyped using the Illumina 1M and the Affymetrix 6.0 arrays. We downloaded genotypes for all the individuals in this dataset from the HapMap project website (http://hapmap.ncbi.nlm.nih.gov/) We removed related individuals and pruned a subset of SNPs based on Linkage Disequilibrium (LD) (*r* ^2^ threshold of 0.3) using the Plink software tool [35] to generate a reduced set of 249,075 SNPs with genotypes for 1198 unrelated individuals. For each population, allele counts were calculated for each SNP using plink (–freq command) and allele frequencies were estimated from the allele counts.

### Simulations

To assess how accurately our method can recover the true admixture coefficients, we simulated admixed individuals using allele frequencies from the HapMap 3 dataset. We simulated an inter continental admixture scenario with admixture between the CEU, CHB and YRI populations. For each individual, the admixture coefficients for the three populations were sampled uniformly at random from a 2-dimensional unit simplex (*x*_1_+*x*_2_+*x*_3_=1) and the genotypes were simulated using the genotype likelihoods defined in Equation 1. We simulated genotypes for 100 individuals and estimated admixture coefficients using our method. For each simulated individual, we used the root mean square error (RMSE) to assess the accuracy of the admixture coefficients estimated by our method. The RMSE was calculated using the following formula:

$$ RMSE (\hat{a},a) = \sqrt { \frac{1}{k} \sum\limits_{i=1}^{k} { (\hat{a_{i}} -a_{i})}^{2}} $$

where *k* is the number of reference populations and *a*_*i*_ is the admixture coefficient associated with population *i*. Results from the simulations showed that iAdmix was able to estimate the admixture coefficients quite accurately with a mean RMSE of 0.0028 (range from 0.0004-0.015).

The simulations utilized the same set of allele frequencies to estimate the admixture coefficients using iAdmix that were used to simulate the individuals. This does not capture the variance in the allele frequencies due to finite sample size of the reference populations. To mimic a realistic setting with noisy population allele frequencies, we sampled genotypes for a finite number of individuals (n = 100) for each population and used the allele frequencies estimated from this sample for admixture analysis using iAdmix. The genotypes were sampled using the true allele frequencies. Results using the noisy allele frequencies indicated that admixture proportions associated with different continents (Europe, East Asia and Africa) can be estimated with high accuracy (mean RMSE = 0.0037) but it is difficult to estimate the admixture coefficients associated with populations within each continental group. For example, we observed that the European admixture component estimated by our method is split between the CEU and TSI populations. This is likely due to the low differentiation between some populations from the same continent (e.g. Fst between the CHB and CHD populations from East Asia is 0.001 while the Fst between the CEU and TSI populations is 0.004 [12]). We also estimated admixture coefficients using the ADMIXTURE program run in supervised mode using the simulated genotypes for 100 individuals per population as the reference clusters. The mean RMSE averaged over 100 simulations was 0.0031, marginally lower than the mean RMSE for our method. Overall, the simulations indicated that our method can estimated admixture coefficients associated with different continental populations with high accuracy.

### Analysis for Mozabite individuals in the HGDP

**Figure 1:**
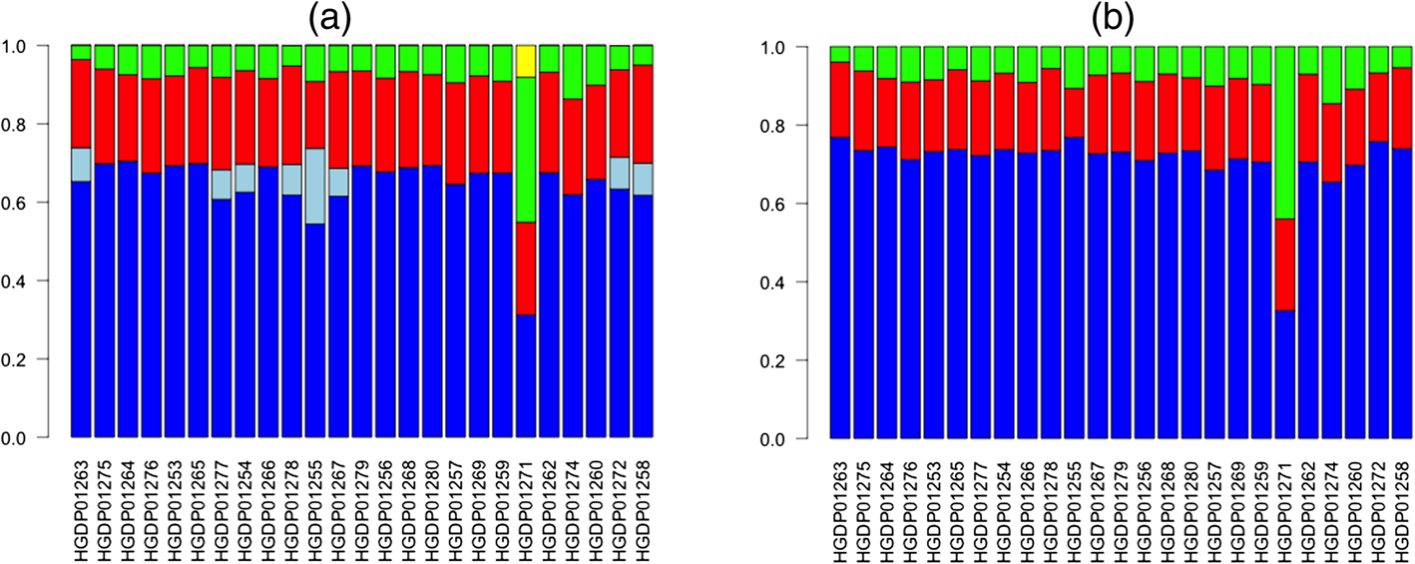
**Admixture proportions for 25 Mozabite individuals.** The coefficients were estimated using allele frequencies from the HapMap reference populations and using two methods: iAdmix **(a)** and ADMIXTURE **(b)**. The population labels are as follows: TSI (blue), CEU (light blue), MKK (red), YRI (green) and LWK (yellow).

To evaluate the ability of our method to estimate admixture coefficients from real data, we analyzed genotype data from 25 individuals from the Mozabite population in the Human Genome Diversity Panel (HGDP) [11]. We downloaded Illumina genotypes at ∼ 650,000 markers for these individuals from the HGDP website and 114,056 of these markers were in common with the reduced set of 249,075 SNPs from the HapMap dataset. We ran our method, iAdmix, on each individual separately using allele frequencies from 8 HapMap populations (the three admixed populations GIH, MXL and ASW were excluded). The admixture estimates (see Figure 1(a)) show that all the individuals are admixed with both European and African components of ancestry. Price et al. [36] analyzed the same set of individuals using their local ancestry inference method, HAPMIX, and estimated that the Mozabite individuals have approximately 78% ancestry from a European-related population and 22% from a population related to sub-Saharan Africa. Our estimates of admixture coefficients are consistent with the local ancestry based estimates.

For comparison, we also ran ADMIXTURE (in supervised mode using the HapMap reference panel of individuals) on the same dataset (see Figure 1(b)). The European and African admixture estimates for each individual were highly consistent between the two methods. For some individuals, the European component of ancestry using our method was split between the TSI and CEU populations. This could reflect one important difference between the two methods in how they use data from reference individuals. Our method finds a maximum likelihood estimate of the admixture coefficients for each individual using the fixed set of allele frequencies. In contrast, ADMIXTURE, in the supervised mode, utilizes data for all individuals (both the reference populations and the individual(s) being analyzed) to estimate the allele frequencies for each cluster or population and maximize the likelihood function summed across all individuals. Therefore, the allele frequencies are determined not only by the genotypes of the reference individuals but also by the individual(s) that are analyzed for admixture. To confirm this, we estimated allele frequencies by running ADMIXTURE twice: (1) using 800 reference individuals simulated using allele frequencies for 8 HapMap populations (100 individuals per population, see previous section) and (2) 800 reference individuals and 1 additional individual with 100% CEU ancestry simulated using the HapMap allele frequencies. Subsequently, we used our method to estimate admixture coefficients for the simulated CEU individual using the two sets of allele frequencies separately. We found that using the first set of allele frequencies, the admixture coefficients for both CEU and TSI were non-zero. In contrast, using the second set of allele frequencies, only the CEU admixture coefficient was non-zero. This was similar to the results observed in the analysis of the Mozabite data and provided an empirical validation of our hypothesis regarding the difference in the admixture coefficients estimated by the two methods.

### Estimating ancestry from DNA sequence reads

**Figure 2:**
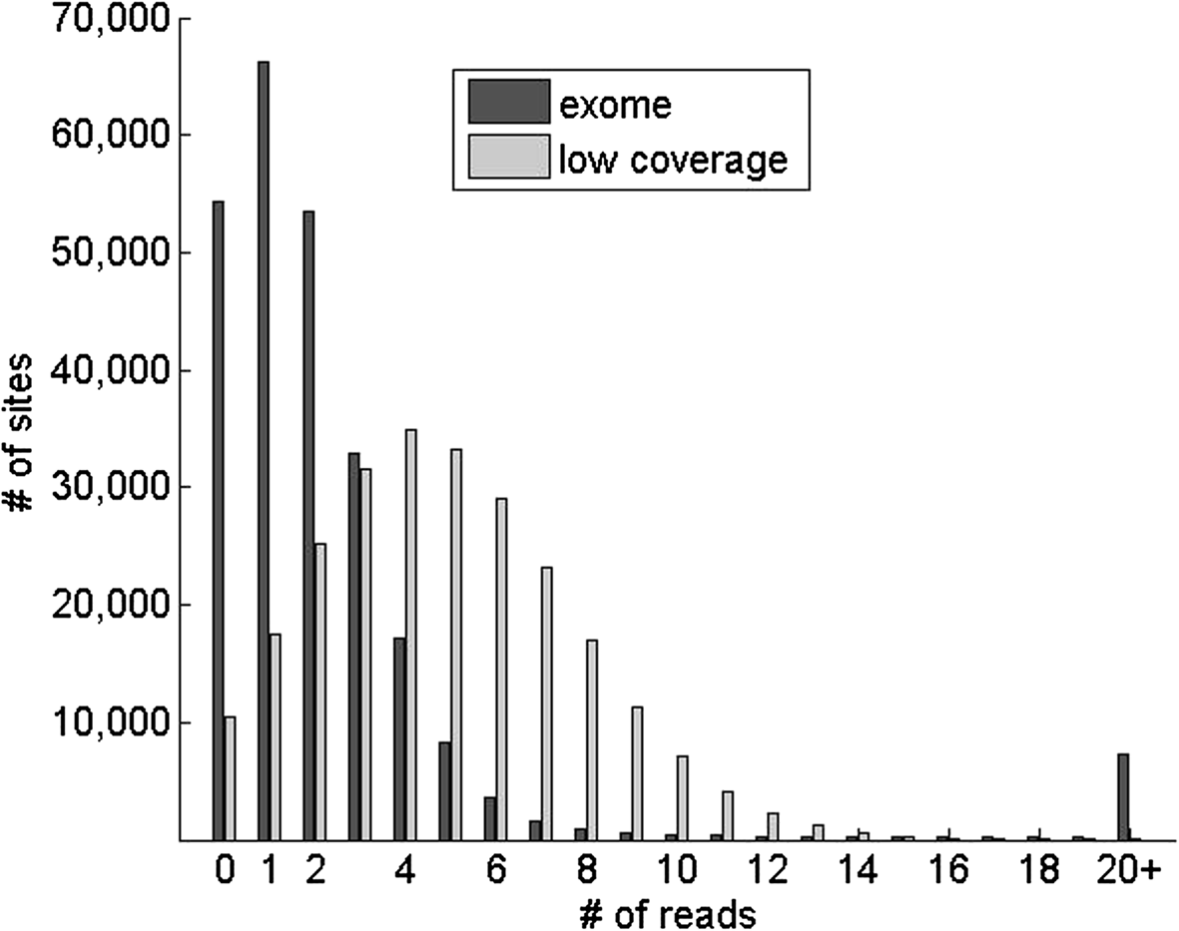
**Distribution of the number of reads covering the 249,075 polymorphic sites in the HapMap3 allele frequency panel using low-coverage whole-genome and exome sequence data from one individual (NA19704) sequenced in the 1000 Genomes Project.**

Next, we assessed the performance of our method on sequence data from the 1000 Genomes Project [18]. For this, we utilized 6 individuals from the ASW population (individuals with African ancestry in SouthWest USA) whose genomes have been subjected to both low coverage whole-genome sequencing and exome sequencing on the Illumina sequencing platform. We downloaded bam files with the aligned sequence reads for the 6 individuals from the 1000 Genomes Project website (ftp://ftp-trace.ncbi.nih.gov/1000genomes/ftp/data/). For each bam file, genotype likelihoods (defined in equation (3)) were calculated at each site in the HapMap3 allele frequency data that had one or more reads covering it. We analyzed the distribution of the depth of coverage across the sites using the reads for one individual (see Figure 2). Interestingly, the exome data had at least one read covering 78.2% of the 249,075 sites. In comparison, 95.8% of the sites had non-zero read depth using the low-coverage data. We calculated admixture proportions using iAdmix for each of the 6 individuals (see Table 1) and summed the admixture proportions associated with population within three continental groups (African, European and East Asian). We observed very high concordance between the admixture proportions estimated using the low-coverage and the exome sequence data (root mean square difference between the two admixture vectors for each individual ranged from 0.003-0.0064). Unexpectedly, one individual (NA19625) was estimated to have significant East Asian related ancestry (16.5%). Analysis of genotype data from this individual carried out in the HapMap project also indicated the presence of East Asian ancestry [12], confirming our results. Overall, these results demonstrate the feasibility of directly estimating ancestry from both whole-genome and targeted sequencing experiments.

**Table 1:** **Comparison of admixture estimates for individuals from ASW population**

**SampleID**	**Data type**	**European**	**African**	**East Asian**
NA19625	lowcov	0.6657	0.1718	0.1626
	exome	0.6672	0.1674	0.1654
NA19700	lowcov	0.8308	0.1692	0
	exome	0.8341	0.1656	0
NA19703	lowcov	0.8554	0.1445	0
	exome	0.8564	0.1437	0
NA19704	lowcov	0.8622	0.138	0
	exome	0.8577	0.1423	0
NA19707	lowcov	0.7397	0.243	0.0173
	exome	0.7354	0.2456	0.0189
NA19701	lowcov	0.8447	0.1313	0.024
	exome	0.8446	0.1286	0.0268

Admixture estimates were calculated using low-coverage whole-genome sequence data (lowcov) and exome sequence data for 6 individuals from the ASW (African-American) population in the 1000 Genomes project.

### Analysis of pooled sequence data

To assess the ability of our method to estimate admixture coefficients from pooled sequence data, we utilized exome sequence data from the 1000 Genomes Project [18] to simulate pools. We downloaded bam files containing exome sequence data for individuals from a European population (Britain, GBR), an East Asian population (Southern Han Chinese, CHS) and an African population (Luhya, LWK). We created four pools by merging the reads from the individual bam files. The first pool contained reads from 20 GBR individuals, the second pool was composed of reads from 19 GBR individuals and 1 CHS individual, a third pool contained reads from 19 GBR individuals and 1 LWK individual, and the fourth pool was comprised of reads from 18 GBR individuals, 1 CHS individual and 1 LWK individual. The rationale for creating these simulated pools was to assess the ability of our method to determine if the ancestry of the individuals in a pool was homogeneous or if one or more individuals in a pool had ancestry from other populations. This would be useful in a case-control association study to identify pools with non-homogeneous ancestry. To mimic the scale of a targeted sequencing experiment, we utilized reads that mapped to chromosome 11 only.

For each pool, we calculated the admixture coefficients using our method and the allele frequencies from the HapMap dataset. To maximize overlap between the sequence reads and the variants in the HapMap dataset, we utilized all genotyped SNPs instead of the LD pruned subset of SNPs.

For the pool with the 20 GBR individuals, only the European populations (CEU and TSI) had non-zero admixture coefficients. For the pool that included reads from a single CHS individual, an East Asian population (CHD) had a non-zero admixture coefficient that was statistically significant (see Table 2). Similarly, we observed a non-zero African admixture coefficient for the pool with one LWK individual and two non-zero admixture coefficients (corresponding to East Asian and African populations) in the pool with 2 non-European ancestry individuals (Table 2). To assess the ability to detect admixture in larger sized pools, we simulated pools with 40 individuals (39 GBR and 1 CHS) and 60 individuals (59 GBR and 1 CHS). Our method was able to detect the presence of East Asian ancestry in the pool with 40 individuals (expected = 0.0257, observed = 0.0295) as well as the pool with 60 individuals (expected = 0.0164, observed = 0.0207). These results demonstrated that our method can reliably detect the presence of individuals with non-European ancestry in a pool of European ancestry individuals using sequence reads from the pool.

**Table 2:** **Admixture coefficients for simulated pools**

**Pool composition**	**European**	**East Asian**	**African**
20 GBR	1.0	0	0
19 GBR, 1 CHS	0.9465	0.0535	0
19 GBR, 1 LWK	0.9653	0	0.0347
18 GBR, 1 LWK, 1 CHS	0.9116	0.0562	0.0323
39 GBR, 1 CHS	0.9705	0.0295	0
59 GBR, 1 CHS	0.9793	0.0207	0

Pools were constructed using exome sequence data from the 1000 Genomes data and the admixture coefficients estimated using allele frequencies from 8 HapMap reference populations.

The ability to estimate admixture coefficients is dependent on the number of variants with genotype information from the sequence reads. For each pool, the number of SNPs that had non-zero coverage was ∼ 72,000 and of these, ∼ 3,300 SNPs had an average coverage of 20 × or greater per individual. To assess the accuracy of estimating admixture coefficients as a function of the number of SNPs, we analyzed the pool with 19 GBR individuals and 1 CHS individual (East Asian admixture coefficient = 0.05) with random subsets of SNPs with varying percentage (5-40%) of the total numbers of SNPs. Not surprisingly, the standard deviation of the admixture coefficient for the East Asian ancestry was high (0.0096 for 50 samples) at 5% and decreased to 0.0032 as the percentage of SNPs used increased to 40% (see Additional file 1: Figure S1).

### Implementation and running time

To optimize the likelihood function, we utilized the open source implementation of the L-BFGS-B algorithm by Zhu and colleagues [37]. The computational complexity for each iteration of the BFGS algorithm is *O*(*n* *k* *p*) where *n* is the number of SNPs, *k* is the number of reference populations and *p* is the pool size. However, the total run time depends on the number of iterations required for the convergence of the BFGS optimization. The BFGS method was run until the difference between successive log-likelihoods was less than 0.00001. The same convergence criterion has been used by previous methods [9]. In all the evaluations using both real and simulated data, the number of iterations for convergence was typically 20-30 and did not exceed 50. We initialized the admixture coefficients with random values between 0 and 1. Empirical evaluation showed that the optimization converged to the same final solution regardless of the initial admixture coefficients.

The main method was implemented in C and the input and output files were processed using Python. To calculate genotype likelihoods for variant sites from BAM files, we implemented a custom program using the Samtools library [38].

Our method analyzes one sample at a time and the average run time per sample for our method (averaged across 100 simulations) was 5.2 seconds for the initial BFGS optimization and 14.8 seconds for the full method including the parsimonious estimation of admixture coefficients. In comparison, the average run time for ADMIXTURE in supervised mode was 87.6 seconds per sample. To assess the ability of our method to estimate admixture proportions associated with a large number of reference populations, we estimated admixture proportions for the Mozabite individuals using allele frequencies at 16,433 SNPs derived from a reference panel of 26 global populations [39]. Our method was able to estimate admixture coefficients with an average run time of 6.4 seconds per individual compared to 57 seconds for a supervised ADMIXTURE run (results not shown). All evaluations were done on a single core of an Intel Xeon processor (2.6 GHz) with 64-bit Linux system.

## Conclusions

In this paper, we have described a computationally fast and efficient method, iAdmix, which can be used to infer global andmixture proportions from genotype or sequence data using a reference set of population allele frequencies. This method employs the BFGS optimization algorithm, which makes it possible to estimate an individual’s admixture proportions from whole-genome genotype data in seconds even in the presence of multi-way admixture. Using simulations, we have demonstrated that our method is able to deconvolute admixture associated with multiple continental populations with comparable accuracy and significantly better speed then existing methods. The increased computational efficiency is the main advance of our method as it allows us to estimate admixture proportions associated with a large number of ancestral populations and also to run iAdmix iteratively in order to obtain parsimonious admixture estimates.

The likelihood model for estimating the admixture proportions assumes Hardy-Weinberg equilibrium (HWE) to calculate the genotype likelihoods. This model can be extended to capture deviations from HWE due to inbreeding [40] and simultaneously estimate the admixture coefficients and the inbreeding coefficient. This may be useful for analysis of individual genomes from populations with some level of inbreeding in order to identify disease causing mutations. Preliminary results indicate that the admixture coefficients are robust to deviations from HWE (results not shown) and we plan to investigate this further in the future.

Another key advantage of our method is that it uses allele frequencies rather than individual genotypes. Therefore it can leverage allele frequencies for populations for which no ‘pure’ or non-admixed exist or are difficult to obtain. For example, Bustamante and colleagues [41] have estimated allele frequencies for Native American populations using local ancestry analysis of populations sequenced in the 1000 Genomes Project that can be used for admixture analysis of Hispanic individuals. The accuracy of ancestry inference by our method relies on the availability of accurate allele frequencies for a large number of reference populations. In this paper, we used allele frequencies calculated from samples collected as part of the HapMap3 project. While an impressive undertaking, the populations contained in this resource are a limited sampling of the global population diversity. A more comprehensive panel would be extremely useful as it would allow for a more meaningful and accurate inference. The 1000 Genomes project is generating sequence and genotype data on more than 25 different populations and once completed, it would be a valuable resource for reference human populations. Many populations have already been sampled by various research groups, and a large number of publicly available genotype datasets exist. The collation of these disparate resources is an important topic for future work.

The described method addresses the problem of estimating the genome-wide average or global ancestry of an individual. In many applications, local ancestry, i.e., the ancestry of a chromosomal segment that has been inherited from an ancestor associated with a single parental population, is of interest. However, this is a difficult problem and existing methods for inference of local ancestry typically consider only two or three ancestral populations [36,42-44]. Our method was motivated by the need for estimating ancestry in sequencing based association studies where global admixture estimates can be used as covariates in association analysis or to exclude outlier individuals. Sequencing data poses new challenges for admixture estimation but also presents opportunities for the development of methods that can exploit information present in sequence data that may be missing in genotype data, e.g. relating to rare or population-specific variants [45]. With the increasing use of high-throughput sequencing technologies, methods such as iAdmix and other recently developed methods [20-22,45] should prove useful for the assessment of ancestry in studies of human genetic variation and disease.

## Additional file

Additional file 1
**Supplementary figure.**
