# Supplementary material for: Fast individual ancestry inference from DNA sequence data leveraging allele frequencies for multiple populations
Source: BMC Bioinformatics. 2015 Jan 16;16:4. doi: 10.1186/s12859-014-0418-7 (PMC4301802; doi:10.1186/s12859-014-0418-7)
Supplement: Additional file 1 — Supplementary figure. [file 12859_2014_418_MOESM1_ESM.pdf]

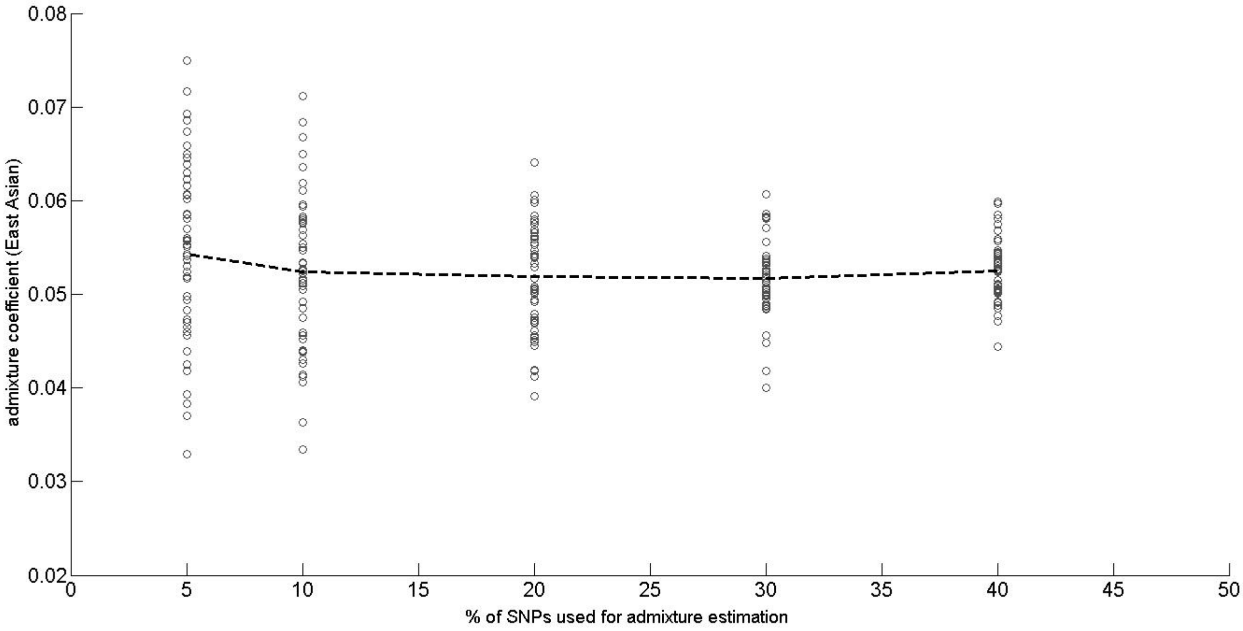

Figure S1: *Variance of the admixture coefficient as a function of the percentage of SNPs used for ancestry analysis.* Admixture coefficients were estimated using iAdmix for the simulated pool with 19 GBR (European) individuals and 1 CHS (East Asian) individual. For each percentage value on the x-axis (5%, 10%, 20%, 30% and 40%), a set of SNPs was constructed by randomly sampling the corresponding percentage of SNPs from the set of all sites with non-zero read depth. This procedure was repeated 50 times for each percentage value. The y-axis is the admixture coefficient for the East Asian ancestry.
